# The pre-vaccination regional epidemiological landscape of measles in Italy: contact patterns, effort needed for eradication, and comparison with other regions of Europe

**DOI:** 10.1186/1478-7954-3-1

**Published:** 2005-02-17

**Authors:** Piero Manfredi, Eugene M Cleur, John R Williams, Stefania Salmaso, Marta Ciofi degli Atti

**Affiliations:** 1 Dipartimento di Statistica e Matematica Applicata all'Economia, Università di Pisa, Via Ridolfi 10, 56124 Pisa, Italy; 2 Department of Infectious Disease Epidemiology, Division of Primary Care & Population Health Sciences, Faculty of Medicine, Imperial College London, Norfolk Place, London W2 1PG, UK; 3 Laboratorio di Epidemiologia e Biostatistica, Istituto Superiore di Sanità, Viale Regina Elena 261, 00198 Roma, Italy

## Abstract

**Background:**

Strong regional heterogeneity and generally sub-optimal rates of measles vaccination in Italy have, to date, hampered attainment of WHO targets for measles elimination, and have generated the need for the new Italian National Measles Elimination Plan. Crucial to success of the plan is the identification of intervention priorities based upon a clear picture of the regional epidemiology of measles derived from the use of data to estimate basic parameters. Previous estimates of measles force of infection for Italy have appeared anomalously low. It has been argued elsewhere that this results from Italian selective under-reporting by age of cases and that the true measles force of infection in Italy is probably similar to that of other European countries. A deeper examination of the evidence for this conjecture is undertaken in the present paper.

**Methods:**

Using monthly regional case notifications data from 1949 to the start of vaccination in 1976 and notifications by age from 1971–76, summary equilibrium parameters (force of infection (FOI), basic reproductive ratio (*R*_0_) and critical vaccination coverage (*p*_*c*_)) are calculated for each region and for each of 5 plausible contact patterns. An analysis of the spectra of incidence profiles is also carried out. Finally a transmission dynamics model is employed to explore the correspondence between projections using different estimates of force of infection and data on seroprevalence in Italy.

**Results:**

FOI estimates are lower than comparable European FOIs and there is substantial regional heterogeneity in basic reproductive ratios; certain patterns of contact matrices are demonstrated to be unfeasible. Most regions show evidence of 3-year epidemic cycles or longer, and compared with England & Wales there appears to be little synchronisation between regions. Modelling results suggest that the lower FOI estimated from corrected aggregate national data matches serological data more closely than that estimated from typical European data.

**Conclusion:**

Results suggest forces of infection in Italy, though everywhere remaining below the typical European level, are historically higher in the South where currently vaccination coverage is lowest. There appears to be little evidence to support the suggestion that a higher true force of infection is masked by age bias in reporting.

## Background

The WHO target of eliminating indigenous measles in Europe by 2007 represents a challenge for public health systems. The requirements for success in this battle are summarised by Gay [1]. Italy, compared with other European countries, is still quite far from meeting these requirements. Here, in contrast with mandatory tetanus, polio, hepatitis B and diphtheria vaccinations, measles vaccination since its initiation in 1976 is only classed as 'Recommended' and has traditionally been characterised by very low coverage, with a national average in 12–24 months old children of only 56% in 1998 [2], and 76% in 2003 [3], despite intensified and supplementary efforts. A further worrying problem is persistent strong heterogeneity in coverage at the regional level (often also within each region). The substantial measles epidemics in Southern Italy in 2002–2003 [4] tragically underlined these points, also confirmed by routine and serological data [5]. In order to confront this state of affairs Italy is embarking on implementation of a Measles National Elimination Plan [6].

In designing an optimal elimination strategy, it is crucial that planners have at their disposal a clear picture of the regional "geography" of the intensity of effort required for measles elimination as a preliminary step for ranking intervention priorities. This is especially in view of the claim that there could be spatial heterogeneity in transmission rates due to the large socio-economical differences existing in the country [7]. However the evaluation of the required elimination effort in terms of critical coverages still largely and necessarily relies on estimates of basic reproduction numbers (also known as basic reproduction ratios) from pre-vaccination data [8].

Here we deal with this pre-vaccination epidemiology of measles in Italy, taking inspiration from two distinct standpoints. The first is a very practical one, i.e. the need to summarise the degree of effort needed for measles elimination in the Italian regions. This is carried out using the fundamental parameters of the basic SEIR (i.e. Susceptible-Exposed-Infected-Recovered) transmission dynamics mathematical model of vaccine preventable diseases[9]: forces of infection (FOI), defined as the per capita annual rate of infection among susceptible individuals), contact or 'Who Acquires Infection from Whom' (WAIFW) matrices (specifying the rates of transmission of infection between and within age groups) due to contacts arising from its own and from other age groups), and basic reproduction numbers (*R*_0_, the mean number of secondary infections which would arise from the introduction of a primary infection in a wholly susceptible population). As primarily shown by Anderson & May [9-11], provided it is possible to estimate them from high quality pre-vaccination data, these parameters allow concise summary of the natural history of a given infection in a given country in the absence of vaccination, e.g. Edmunds *et al*. [8] for the epidemiology of measles, mumps and rubella (MMR) in Europe.

Edmunds *et al*. [8] have shown that pre-vaccination patterns of measles (and mumps) in European countries were broadly similar, suggesting it may be possible to use parameter values estimated from other countries with good-quality pre-vaccination data to model measles (and mumps) in countries with no or poor infection data. The case of Italy was however more puzzling than that of the other countries. On the one hand they found that the FOI for measles (but also for mumps) computed from Italian national case notifications data was greatly different from other available European FOIs. On the other hand they computed the FOI implicit in the data of Santoro *et al*.[7] - the sole pre-vaccination measles sero-survey in Italy - finding figures that, at least for the youngest age groups (0–4), were in agreement with those summarising infection experience in European countries with good infection data, [8] - here referred to as "EURO" FOIs. They conjectured that Italian data suffered from strong selective under-reporting by age, and concluded: "It is tempting to dismiss FOI estimates from Italian case notifications data as the serological data are likely to be more robust". We believe, however, that this conclusion relies more on the generally assumed greater reliability of serological data compared to case reports, rather than on a full demonstration. Indeed, the FOI they estimated from the data of Santoro *et al*. [7] for school age children (age 5–10) is just 50% of the corresponding EURO FOI, i.e. even smaller than that estimated from case reports. We believe therefore that it would be prudent and worthwhile to try to obtain further insight into this problem. The Edmunds *et al*. [8] paper provides therefore the motivation for our second, more theoretical, question of whether or not the true FOI acting in Italy during the pre-vaccination era is homologous to the EURO FOI, as they suggest? The implications are relevant especially for the purposes of modelling which may be used to inform policy: is it advisable simply to rely on this EURO FOI, or does prudence dictate that one should consider also other possibilities? In what follows we use the term "EURO conjecture" to denote the suggestion of Edmunds *et al*.[8] that i) the Italian FOI would indeed be essentially homologous to the EURO FOI, and ii) Italian case notifications data simply camouflage this fact thanks to broad selective under-reporting. To shed more light on these issues, we have analysed more deeply Italian case notifications data by looking at patterns at several spatial levels.

First we have systematically looked at the structure of the FOIs (and contact patterns, basic reproduction numbers, etc) for all the Italian Regions and Provinces. Since the national datum used by Edmunds *et al*. [8] to compute the Italian FOI, was obtained by pooling regional data in presence of strong spatial heterogeneity in under-reporting (documented in Williams *et al*. [12]), a deeper investigation of spatial patterns of infection could reveal the existence of some between regions heterogeneity in age-related transmission rates that could be an indicator of a higher force of infection (or indeed perhaps suggest the presence of selective under-reporting by age). It is indeed quite conceivable that true heterogeneity might exist within Italy as a result of contrasts between North and South in terms, for example, of family size (including that of the extended family), patterns of shared childcare and schooling, impact of climate on time spent indoors and out, etc.; however in the absence of systematic community based investigation such ideas must remain in the realms of speculation.

**Figure 1:**
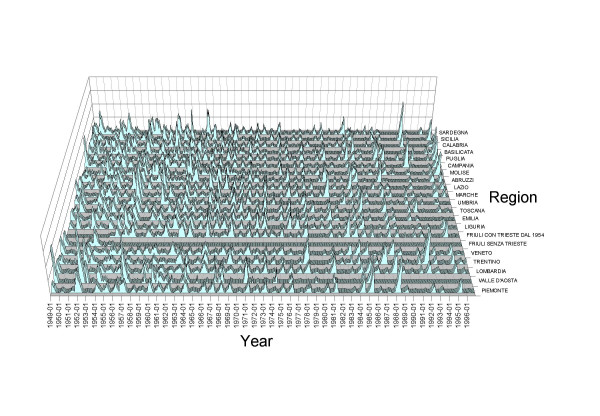
**Measles cases notifications in Italy.** The regionally heterogeneous monthly pattern of Italian measles cases reported for the period 1949-1996 (NB Two time series are shown for Friuli as the city of Trieste was not incorporated into the region until its post-war status within Italy was resolved in 1954)

**Figure 2:**
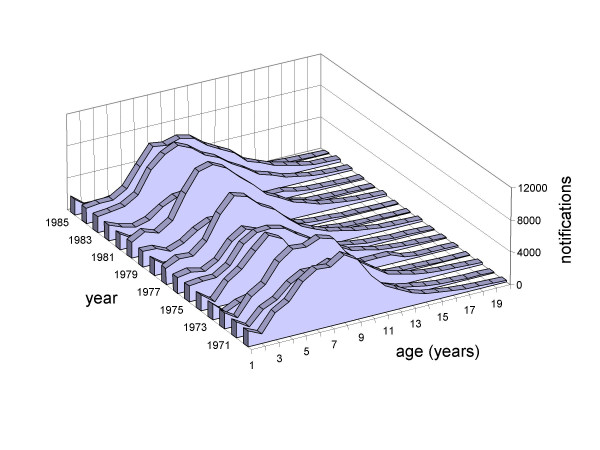
**Measles cases notifications in Italy.** Annual age distributions of measles case reports from 1971 (the first year in which notifications were recorded by year of age rather than age band) through to 1986 (10 years after the start of measles vaccination in 1976, albeit at very low coverage).

Second, we carried out a systematic time series analysis of measles periodicities in the Italian regions during the pre-vaccination era (Fig 1 &2). Our feeling here is that only under exceptional circumstances can selective under reporting by age mask true time patterns of incidence.

Third, we add some results from our modelling work on measles in Italy.

The results suggest that i) forces of infection estimated from case reports of measles in all the Italian regions are systematically and significantly lower compared with the EURO FOI with little, or only moderate, between regions heterogeneity; ii) regional periodicities mostly suggest a longer (usually 3 years or more) inter-epidemic period compared to England & Wales, a fact which is consistent with lower transmission (and which, together with the lower FOIs, suggests lower vaccination coverage might suffice to achieve control and elimination); iii) predictions from a mathematical model using the EURO force of infection, even compared with those based on a FOI estimated from Italian case reports, poorly match the 1996/1997 Italian serological data [13].

## Methods

The geographic analysis of the epidemiology of vaccine preventable diseases is made difficult by complex correlations between local dynamics. A first, though clearly partial, step is characterization of local infection patterns by treating spatial sub-areas as autonomous epidemiological units. Two measures are used here to characterise the regional (i.e. local) "landscape" of measles in Italy in the pre-vaccination era: summary parameters from the SEIR model for vaccine preventable disease, and summary periodicities from time series analyses of measles incidence.

### Age patterns of infection, reproduction numbers and critical vaccination coverage

From the pre-vaccination age distribution of cases we computed, for each region, summary equilibrium parameters from the SEIR model: i) forces of infection, FOI; ii) mixing or contact ("who acquires infection from whom", WAIFW) matrices; iii) basic reproduction numbers, *R*_0_; and iv) related critical vaccination coverages, *p*_*c*_, the proportion of a population needed to be successfully immunised at age zero with a 100% effective vaccine in order to eliminate the infection, where *p*_*c *_= 1-1/*R*_0 _(i.e. whereas in a wholly susceptible population one primary case will on average successfully transmit to *R*_0 _contacts, in the case when the proportion already immune is greater than *p*_*c *_transmission will succeed for less than *R*_0_(1-*p*_*c*_) = *R*_0 _(1- [1-1/*R*_0_]) = *1.0 *contacts) [9].

For comparison purposes we used the same age groups as in Edmunds *et al*. [8], i.e. 0–1 years, 2–4 yr., 5–10 yr., 11–17 yr., 18+ yr. The basic reproduction numbers [14] were computed for several plausible mixing matrices.

### Mixing (WAIFW) matrices

Mixing matrices (Table 1) are used in standard infectious disease modelling [9] to summarise age patterns of contacts between susceptible and infected individuals; the generic element *β*_*ij *_summarises the risk of acquiring infection for a susceptible individual in age group *i *due to contacts with infective individuals aged *j*. When estimating from age-structured data (e.g. serology or case reports), a mixing matrix with *m *age groups can have at most *m *distinct elements [9]. In the simplest type of mixing, i.e. homogeneous mixing, the *β*_*ij *_entries are independent of age (*β*_*ij *_= *β *for all *i,j*). Other types of mixing matrix are considered in the paper and are listed below. However not all forms of mixing are compatible with a given force of infection. As noted by Anderson & May [9], a mixing matrix can be *non feasible *for the given FOI (i.e. it may yield negative values for some of the *β *coefficients) and in such circumstances "the chosen matrix is inappropriate to the observed age dependence in the FOI". Hethcote [15] further pointed out that not all feasible mixing matrices are "acceptable", in that they could lead to numbers of contacts between individuals outside plausibility bounds; inspection of the ensuing mixing matrices is thus necessary to avoid trivial results. Hethcote also proposed a "plausibility criterion", based on a postulated "preference for assortativeness", which is simple to use for preferred matrices (type PREF below).

**Table 1:** Some of the types of mixing matrices used in the paper. Three of the mixing matrices discussed in Methods: a) fully assortative mixing (RDIAG), b) "Realistic" assortative mixing (DIAG) and c) the default mixing matrix (DEF) of Edmunds *et al *(2000). Succeeding rows and columns represent the age groups 0–1 year, 2–4 years, 5–10 years, 11–17 years and 18+ (e.g. for DIAG *β*_3 _is the element corresponding to contacts between those in the 5–10 year age group and their peers in the same age group)

a) *DIAG*						b) *RDIAG*						c) *DEF*				
*β*_1_	*0*	*0*	*0*	*0*		*β*_1_	*β*_5_	*β*_5_	*β*_5_	*β*_5_		*β*_1_	*β*_1_	*β*_1_	*β*_1_	*β*_5_
*0*	*β*_2_	*0*	*0*	*0*		*β*_5_	*β*_2_	*β*_5_	*β*_5_	*β*_5_		*β*_1_	*β*_2_	*β*_4_	*β*_4_	*β*_5_
*0*	*0*	*β*_3_	*0*	*0*		*β*_5_	*β*_5_	*β*_3_	*β*_5_	*β*_5_		*β*_1_	*β*_4_	*β*_3_	*β*_5_	*β*_5_
*0*	*0*	*0*	*β*_4_	*0*		*β*_5_	*β*_5_	*β*_5_	*β*_4_	*β*_5_		*β*_1_	*β*_4_	*β*_5_	*β*_3_	*β*_5_
*0*	*0*	*0*	*0*	*β*_5_		*β*_5_	*β*_5_	*β*_5_	*β*_5_	*β*_5_		*β*_5_	*β*_5_	*β*_5_	*β*_5_	*β*_5_

### Matrix types considered

#### 1) Fully assortative mixing (matrix DIAG, Table 1(a))

Individuals of a given age are assumed to mix only with individuals of the same age, yielding a diagonal matrix. Given a specific FOI it is the form of mixing allowing *R*_0 _to achieve its upper bound [16].

#### 2) Realistic assortative mixing (RDIAG, Table 1(b))

This matrix (termed "Diagonal" in Edmunds *et al*. [8], see also [17]) has the same diagonal elements as the fully assortative one, but some mixing across age groups is also possible (i.e. non diagonal elements are greater than zero). Here mixing across age groups is assumed to be at the same rate (*β*_5_) as that between adults [8].

#### 3) Default mixing (matrix DEF in Table 1(c))

This matrix emphasises transmission between school age groups [8].

#### 4) Proportionate mixing (PM)

Under PM [15] contacts occur at random, thus implying a larger probability of meeting more socially active individuals. The entries of the PM matrix have "multiplicative" form *β*_*ij *_= *b*_*i*_*b*_*j *_*j *= 1,...*m*.

#### 5). One-parameter preferred mixing (PREF)

The PREF matrix is a single parameter (*h*) weighted average of proportionate (PM) and fully assortative (DIAG) mixing: *PREF *= (1 - *h*) * *DIAG *+ h* *PM*, 0<*h*<1 [15], representing a contact pattern which can be split into a selective (i.e. non random) component (here mixing with individuals of the same age) and a random one. The *PREF *matrix has (*m+1*) distinct entries: the extra parameter *h *is usually estimated "ad hoc".

The matrices RDIAG & DEF are defined "*ad hoc*", though they have a behavioural basis, whereas DIAG & PM are limit cases (though in distinct senses), and hence PREF is a weighted average between two limit cases. There is thus no clear relationship between, for instance, RDIAG & DEF on the one hand and PREF on the other. For this reason, we explored all forms that have been reported in the paper.

### Periodicities of time series of measles incidence

In contrast to the spatially well synchronised biennial England & Wales oscillation, visual inspection of Italian regional data did not suggest clear common patterns of oscillation or hence of the inter-epidemic period. Thus investigation of periodicities in regional measles incidence became necessary to provide satisfactory characterisation of the inter-epidemic period. Though incidence data might be seriously affected by under-reporting, recent work [12] suggests that, as long as we are concerned with the pre-vaccination period, overall under-reporting rates in the Italian regions seem to have remained fairly constant over time. Thus the available time series should nonetheless represent a sufficiently reliable picture of the regional dynamics of measles.

The cyclical behaviour in the monthly incidence time series of measles for each Italian region in the pre-vaccination period 1949–1976 was analysed in the frequency domain (first used for childhood diseases by Anderson *et al*. [18]) with special attention being given to the long term cycle.

A drawback of periodogram analysis is the assumption that cyclical components of frequencies depend on the length of the observed series, i.e. that they are integer multiples of *2π/T *(*T *= series length); this is the basis of "harmonic" analysis. Such analysis may not identify exactly cyclical components where true frequency falls between two "harmonic" frequencies, e.g. it may suggest periodicity of either 3 or 4 observations when true periodicity lies between these values and hence is not identifiable exactly with this procedure. This problem may be overcome with "non-harmonic frequency domain" analysis where dependence of periodicities of cyclical components on series length is relaxed. Here the following procedure, as proposed in [19], is used for identifying true cyclical components: after log transformation to stabilise variances, and de-trending, using a deterministic function of time, the true frequency of a cyclical component, denoted by λ, was estimated, as in [19], by minimising, with respect to *λ*, the quantity



where *x*_*t *_denotes the detrended series (a starting value for *λ *was obtained by examining a non-parametric estimate of the spectral density function; significant non-harmonic functions can be estimated in this way and deleted, if required, in a stepwise manner beginning with the frequency corresponding to the largest spectral density ordinate).

The results from the time series analysis were also compared with the prediction from the homogeneous mixing SEIR model that where the sum *K *of the expected duration of the latent and infectious states is short compared to the life of the host (as is the case with measles), disease incidence will have a long-term oscillation around its endemic equilibrium with the period given, to an excellent approximation, by [9], where *A *is the average age at infection and *K *can be taken to be 14 days in the case of measles.

Analyses described above were carried out for all Italian regions (Valle d'Aosta, a very small northern Region with few reported cases per year was aggregated with neighbouring Piemonte).

### Data

In addition to published official Italian data on births, and birth and death rates, monthly measles case reports were provided by the Istituto Nazionale di Statistica (ISTAT) for the period from the first available year, 1949, to 1976, together with regional age structured measles case reports from the first available year, 1971. Pre-vaccination FOIs at the regional level were estimated from data for the time window 1971–76, which encompassed about two full three-years long epidemic cycles (inclusion also of the first few years in the post-vaccination window, when vaccine uptake was known to be extremely small, resulted in no significant change).

## Results: the pre-vaccination landscape of measles in Italy

### Regional patterns of incidence over time

The spectral densities of the pre-vaccination (1949–76) Italian regional time series of measles incidence indicate, besides a well-pronounced annual cycle, the presence of a less pronounced longer term cycle of varying length, which is in contrast with the sharp biennial oscillation in England & Wales. Given the importance of the long term oscillation which is taken as representing the true "inter-epidemic period", the non-harmonic estimator was calculated (after de-trending each series using a function of time, and de-seasonalising, using monthly dummies,).

Table 2 reports for each region: i) the average age of cases in the pre-vaccination period (*A*); ii) the non-harmonic estimate *T*_*O *_of the period of the long-term oscillation; iii) the length of the inter-epidemic period from the homogeneous SEIR model via formula for *T *given in the Methods section.

**Table 2:** Average age at infection (*A*_15_) and inter-epidemic period (T) in the Italian regions. Results arising from the time series analysis of regional measles notifications data for the period 1949–76 (N = North, C = Centre, S = South.)

	***A*_15 _*(years)***	***T from non harmonic estimate (T_O_) (years)***	***T from SEIR model (T) (years)***
Piemonte & Valle Aosta (N)	6.34	2.39	3.08
Lombardia (N)	5.70	2.37	2.94
Trentino (N)	5.75	2.85	2.94
Veneto (N)	5.87	3.21	2.98
Friuli (N)	5.98	2.77	2.98
Liguria (N)	6.92	5.34	3.22
Emilia (N)	6.24	3.33	3.06
Toscana (C)	7.30	2.85	3.29
Umbria (C)	6.81	3.40	3.21
Marche (C)	6.63	3.27	3.16
Lazio (C)	6.34	3.23	3.08
Abruzzo (S)	6.46	3.40	3.13
Molise (S)	6.19	5.88	3.04
Campania (S)	5.64	3.03	2.90
Puglia (S)	5.20	3.79	2.79
Basilicata (S)	5.43	3.74	2.82
Calabria (S)	5.63	3.45	2.90
Sicilia (S)	5.47	3.14	2.85
Sardegna (S)	5.70	3.79	2.92
North	6.09	2.35	3.02
Centre	6.78	3.27	3.22
South	5.65	3.68	2.90
Italy	6.18	2.35	3.06

Values of *T*_*O *_in table 2 show that, apart from a few Northern regions with a long term cycle with a period below 3 years (the shortest period, 2.4–2.5 years, being observed in Piemonte and Lombardia), most Italian regions have a three-year, or greater, long term oscillation (though a note of caution must be sounded with regard to the 5 year period observed in Molise, a very small isolated region). In comparison, values of *T *predicted by the SEIR model range from a minimum of 2.8 (Puglia) to 3.3 years (Tuscany). The agreement between *T *and *T*_*O *_in some cases is not very good: North-Eastern regions show a higher average age at infection, *A*, compared to Southern regions and yet the shorter inter-epidemic periods in the former compared with the latter, as suggested by the time series analysis, would imply the reverse.

Compared to England & Wales there is also a surprising lack of synchronisation between regional cycles in the pre-vaccination period. A cross-spectral analysis suggests limited correlation between pre-vaccination long term cycles; coherencies greater than 0.7 were observed, as expected, only for a few neighbouring regions such as Emilia-Romagna and Marche, Marche and Umbria, Friuli and Trentino, Lombardia and Piemonte, Liguria and Piemonte

### The structure of the force of infection at the regional level

**Figure 3:**
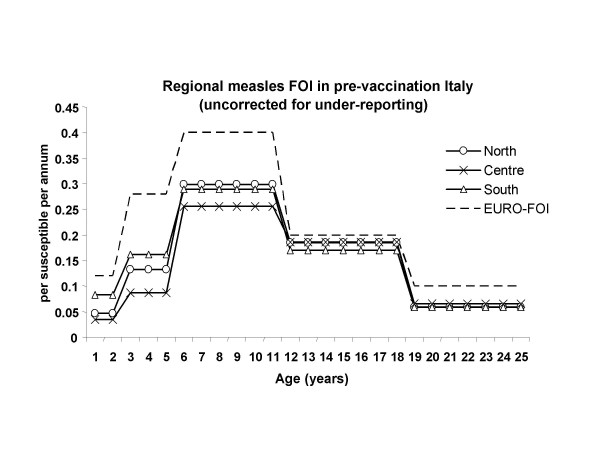
**Pre-vaccination forces of infections in the Italian regions. **Age-related pre-vaccination forces of infection (FOI) estimated from case notifications for North, Centre and South divisions of Italy compared with the "EURO" FOI estimated by Edmunds *et al *[8]

Pre-vaccination age-distributed forces of infections in the Italian regions (we report a sample of results) can be well summarised by 3 clusters, North, Centre, South. These are shown in Fig. 3 together with the EURO FOI [8] which displays a similar qualitative pattern with age (single-humped, peaking in the "elementary school" age group 5–10, etc.), although the "Italian" FOIs exhibit surprisingly lower levels amongst pre-secondary school (< 11 years) age groups (with the exception, perhaps, of the very youngest age group in Southern Italy). Of the three Italian clusters the FOI for the two youngest age groups is highest in the Southern regions, and lowest in the Central regions. Relatively lower FOIs persist in Central regions in the elementary school age group (5–10 y.) compared with those in the North and South which resemble each other. Finally, in the two highest age groups, FOIs are similar, though Central and Northern regions now have marginally higher FOI values than the South.

The average age at infection *A *(computed over the restricted support 0–18 yr. age group, as more robust than the overall average age), that coarsely summarises the force of infection, shows similar spatial patterns. For instance *A *is systematically smaller in the South (little above 5 years), around 7 years in the Centre, and takes intermediate values in the North (Table 2, second column).

From the regional FOIs we also computed a national FOI corrected for under-reporting ('Italy-UR' in Table 3) using the under-reporting factors estimated in Williams *et al*.[12] which suggested great heterogeneity in reporting rates, the higher rates being observed in the Northern Italian regions (around 10–12% in the pre-vaccination era) and the smaller in the South (as low as 2% in Campania!). Conceivably the concurrence of spatial heterogeneity in overall rates of under-reporting with spatial heterogeneity in age-related transmission rates might be a factor responsible for a selective age-bias in the national age distribution even in absence of selective under-reporting by age, because it weights incorrectly the regional cases that are pooled into the national datum. Fig. 3 suggests however that the quantitative impact of the correction for under-reporting is likely to be rather small.

**Table 3:** Measures indicating the size of the task of eliminating measles in the Italian regions. Estimates for basic reproduction numbers (*R*_0_) and critical age zero routine vaccination coverages (*p*_*c*_) for measles elimination in the Italian regions. Results in column pairs correspond to different assumptions on contact patterns. (NA = contact matrix non admissible; * = contact matrix admissible only after FOI redefined on adult age group; N = North, C = Centre, S = South.)

**Type of mixing pattern**	**Homogeneous**		**Default**		**Realistic assortative**		**Proportionate**		**Preferred mixing *ε *= 0.9)**	
	**R_0_**	**P_C _age 0**	**R_0_**	**P_c_age 0**	**R_0_**	**P_c _age 0**	**R_0_**	**P_c _age 0**	**R_0_**	**P_c _age 0**

Piemonte & Valle D'Aosta (N)	11.83	0,92	NA	NA	11.8*	0.92	4.09	0.76	4.30	0.77
Lombardia (N)	13.16	0,92	6.2	0.84	16.1*	0.94	4.80	0.79	5.18	0.81
Trentino (N)	13.05	0,92	5.4	0.81	16.0*	0.94	4.50	0.78	4.82	0.79
Veneto (N)	12.77	0.92	5.5	0.82	17.6*	0.94	4.77	0.79	5.13	0.81
Friuli V.G. (N)	12.53	0.92	4.9	0.80	13.1*	0.92	4.05	0.75	4.32	0.77
Liguria (N)	10.83	0.91	NA	NA	10.0*	0.90	3.73	0.73	3.95	0.75
Emilia (N)	12.01	0.92	NA	NA	17.5*	0.94	4.41	0.77	4.69	0.79
Toscana (C)	10.27	0.90	NA	NA	9.8*	0.90	3.60	0.72	3.79	0.74
Umbria (C)	11.01	0.91	NA	NA	8.5*	0.88	3.62	0.72	3.81	0.74
Marche (C)	11.32	0.91	NA	NA	10.6*	0.91	3.83	0.74	4.06	0.75
Lazio (C)	11.83	0.92	NA	NA	11.8*	0.92	4.06	0.75	4.27	0.77
Abruzzo (S)	11.60	0.91	NA	NA	14.6*	0.93	4.64	0.78	5.07	0.80
Molise (S)	12.11	0.92	NA	NA	13.2*	0.92	5.68	0.82	6.62	0.85
Campania (S)	13.30	0.92	7.5	0.87	16.3	0.94	5.91	0.83	6.89	0.85
Puglia (S)	14.43	0.93	7.80	0.87	20.1	0.95	6.01	0.83	7.14	0.86
Basilicata (S)	13.82	0.93	6.7	0.85	19.8	0.95	5.19	0.81	5.86	0.83
Calabria (S)	13.32	0.92	6.1	0.84	18.5	0.95	5.07	0.80	5.62	0.82
Sicilia (S)	13.71	0.93	7.5	0.87	16.6	0.94	5.91	0.83	6.98	0.86
Sardegna (S)	13.17	0.92	6.3	0.84	16.6	0.94	5.29	0.81	5.93	0.83
North	12.32	0.92	5.2	0.81	14.9*	0.93	4.38	0.77	4.66	0.79
Centre	11.05	0.91	NA	NA	10.4*	0.90	3.76	0.73	3.96	0.75
South	13.27	0.92	6.6	0.85	16.4	0.94	5.45	0.82	6.19	0.84
Italia	12.14	0.92	5.2	0.81	13.2*	0.92	4.33	0.77	4.64	0.78
Italy-UR	12.93	0.92	6.0	0.83	14.3	0.93	5.04	0.80	5.57	0.82
EURO	17.05	0.94	9.6	0.90	29.3	0.97	7.14	0.86	8.95	0.89

A further point worth of consideration in Fig. 3 is that, in contrast to the EURO FOI, in the North and Centre the FOI in the highest age group is higher than the FOI in the youngest (a fact shared by most regions in the two clusters, but not occurring in the Southern regions and which occurs regardless of how we define the last age group). This suggests a greater relative importance of contacts between adults (provided one can exclude the effects of poor reporting).

### Mixing matrices, reproduction ratios and required effort for measles elimination

From the values of the FOI we computed mixing-WAIFW matrices. As previously noted, a problem with the computed Italian FOIs is that risk of infection among adults individuals (i.e. age group 18+) is, with the exception of Southern regions, higher than in the "young" (0–1 yr.). A verifiable consequence of this fact, is that "realistic assortative" mixing matrices (RDIAG) can be non-feasible (they can yield negative coefficients), and this indeed happens in all Central and several Northern regions. In other words, such mixing is not compatible, Southern Regions apart, with observed forces of infection. This may be a problem because "realistic assortative" is the mixing pattern that provides under a EURO-type FOI, the upper bound of "plausible" values of *R*_0_, a measure of critical importance from the perspective of disease control. For purposes of comparability with Edmunds *et al*. [8] we therefore arbitrarily redefined the value of the force of infection in the oldest age group in order to recover, for all Italian regions (and not only the Southern ones) the "EURO" shape (i.e. a FOI having its smallest value in the adult age group). In this manner we obtained feasible and "plausible" "realistic assortative" mixing matrices for all Italian Regions. The results are summarised below (Table 3 gives a synoptic view; we have omitted for sake of brevity the outputs of mixing computations and instead report the more easily interpretable values of reproduction ratios and critical vaccination coverages); the results from "fully assortative mixing" are omitted as leading to trivially high values of *R*_0 _:

#### 1. Homogeneous mixing

Values of *R*_0 _(computed as *R*_0 _≅ *L*/*A *where *L *is the expectation of life, taken as 75 years by assuming type 1 mortality and ignoring regional variability, and *A *the average age at infection in the pre-vaccination era, Table 2) range between a minimum of about 10 (Toscana) up to a maximum around 14.5 in Puglia (17.5 under EURO).

#### 2. Realistic assortative mixing (RDIAG)

This mixing yields an *R*_0 _around 29 under EURO [8]. Thanks to our correction for the adult age group all Italian Regions yield fully admissible transmission rates; the corresponding *R*_0 _values are in the range 13–20 in the South (critical coverages ranging 95–97%) and 8.5–12 in the Centre (critical coverages 88–92%), with intermediate values in the North.

#### 3. Default mixing (DEF)

It happens that this mixing pattern is never admissible for all Central Regions and for some Northern ones, as it can yield negative transmission rates when, as is the case in Central Italy, the FOI is rather low in the youngest age groups. Default mixing is, on the contrary, admissible in all Southern regions where *R*_0 _and critical coverages happen to be systematically higher. Critical coverages range from 80% in the North up to 88% in the South (the reference EURO value being 90%).

#### 4. Proportionate mixing (PM)

PM mixing matrices (always admissible by definition) are the type of mixing [15,17] yielding the lower bound of "plausible" values of *R*_0_. Predicted critical coverages ranged from 72% in the Centre, with intermediate values in the North, up to 83% in the South (EURO = 86%, Table 2). Nevertheless inspection of the matrices does reveal for all regions (also for EURO) the presence of implausible relationships between groups, e.g. a significant level of disassortativeness.

#### 5. Preferred mixing (PREF)

We computed preferred matrices by tuning the "preference for assortativeness" parameter *h *starting from *h = 1 *(proportionate mixing) and then [15] progressively decreasing *h *until "minimally plausible" contact rates were achieved. Essentially, for all Italian regions (also for EURO), use of a moderate degree (*h = 0.9*) of assortativeness allowed the "minimal plausibility" threshold to be surpassed. This led, compared to the case of proportionate mixing, to increases of 1% to 3% in the corresponding critical coverages (Table 3). These figures should be considered as providing more reliable lower bounds on *R*_0 _than does Proportionate mixing.

An issue is the degree of assortativeness, *h*, which would lead to the preferred mixing pattern closest to reality (here we only considered the degree guaranteeing a "minimally plausible" contact pattern, according to Hethcote [15]). Although one-parameter preferred mixing provides a flexible family of mixing patterns, it is still too inflexible to provide realism, as it assumes the same degree of preference for assortativeness in all age groups. For instance considering schooling as the major source of assortativeness in younger age groups, in pre-vaccination Italy a much smaller proportion, compared to the present, of boys in the youngest age group were attending crèche schools so that a major potential source of their assortativeness was probably absent. This would therefore suggest that at least two "preference for assortativeness" parameters (*h*_1_,*h*_2_, say) may be needed to describe Italian pre-vaccination infection patterns.

This also leads to the nub of the real gain provided by preferred matrices (just because they are "many") compared to "ad hoc" behavioural matrices. One could ask whether Hethcote's [15] preference for an assortativeness criterion is meaningful for mixing matrices which do not conform to the one-parameter "preferred mixing" scheme. Using this criterion of "preference for assortativeness", in most cases, "default" mixing would be discarded, and indeed, under default mixing (quite apart from the over small degree of assortativeness postulated for the youngest age group), most Italian regions do not meet this criterion for age groups 2–4 and 5–10. Nevertheless, from other perspectives, "default" is one of the most "reasonable" among behavioural mixing matrices. More generally, any answer to such questions is complex, as the problem of how to assess whether one arbitrary given mixing matrix is "better", or simply more plausible, than another remains unresolved. This problem is not simply an academic one: the predicted critical coverage for measles at age zero under the EURO FOI ranges between 86% under proportionate mixing (PM) and 97% under realistic assortative (RDIAG) [8]. Using the Hethcote [15] criterion one can marginally increase the lower bound up to 87.5% by replacing proportionate mixing (unacceptable under Hethcote criterion) with the corresponding "minimally acceptable" preferred matrix (PREF), but the extent of the uncertainty that remains is hardly less significant.

### A modelling result

Our analysis of the pre-vaccination force of infection of measles in Italy formed a step toward developing a mathematical model for the transmission dynamics of measles in Italy. The model has the following features:

i) it provides bounds for the uncertainty surrounding the estimate of the FOI (Table 4), by taking the "EURO" FOI as upper bound, and as a lower bound the FOI (denoted as Italy-UR in Table 2 & Figure 3) estimated from Italian case reports by correcting regional figures using the estimates of under-reporting in Williams *et al*. [12];

**Table 4:** Force of infection estimates. Estimates of EURO force of infection (FOI) and that from Italian case notifications; computations were based on standard techniques described by Anderson & May [9].

	**Force of infection/FOI (% / year)**				
**Age group (years)**	0–1	2–4	5–10	11–17	18+
**Italy-UR**	7	15	31	19	6
**EURO**	12	28	40	20	10

ii) it incorporates "realistic" demography in order to mirror the broad process of population ageing observed in Italy in the past 20 years (and which would be observed in the future if the present state should continue) as a consequence of the onset of sustained low fertility;

iii) it considers two distinct options as regards interaction between the demography and the epidemiology: the first (D1) assumes no effects on transmission arise as a consequence of population ageing; the second (D2) assumes low fertility and population ageing could lead to a significant decline in transmission (e.g. a decline in the FOI), via proxy mechanisms such as a substantial decline in intra-family transmission which might occur as a consequence of contraction in average family size.

**Figure 4:**
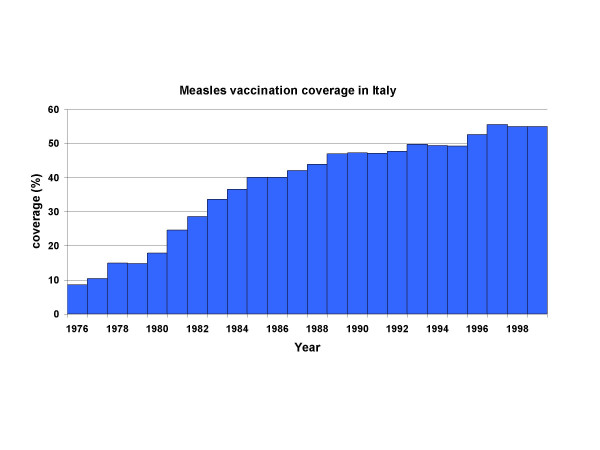
**The reconstructed time profile of measles routine vaccination coverage.** This plausible profile of vaccination coverage was reconstructed from the very limited available national and regional data.

By crossing the two FOI assumptions, EURO and UR (i.e. Italy-UR), with the two assumptions D1, D2 we obtain four assumptions: EURO/D1, EURO/D2, UR/D1, UR/D2. The model simulations were carried out by adopting for the post-vaccination era what we considered the "best" approximation for the profile of routine vaccination coverages (Fig. 4), obtained by combining data collated from the few available regional profiles with the few available national data, such as the 1998 vaccination survey [2].

**Figure 5:**
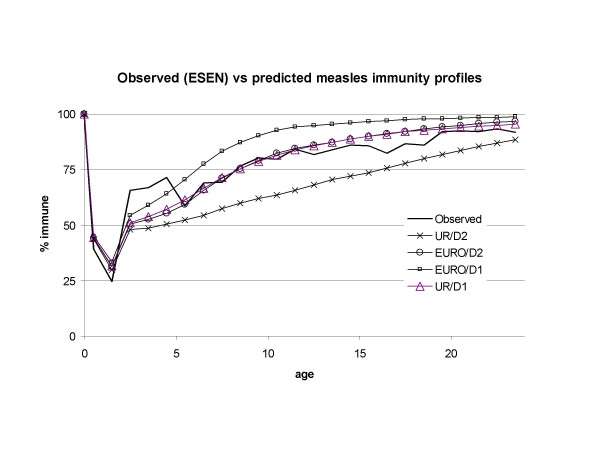
**Observed (ESEN) vs predicted measles immunity profiles.** The serological profile from the ESEN survey is compared with model projections assuming the EURO force of infection of Edmunds *et al *[8] or the FOI estimated from Italian pre-vaccination case notifications but corrected for under-reporting (UR). In each case one of two assumptions is made: i) that the process of population ageing predicted for Italy has no effect upon transmission (D1) or ii) that low fertility and population ageing could lead to significant decline in transmission (D2).

Fig. 5 reports the immunity profile derived from the seroprevalence data obtained by the national survey conducted in 1996–97 (3,182 samples collected from residual sera from routine laboratory testing, in 18/20 Italian regions [13]) compared with the immunity profiles predicted by the model for the same years under the four assumptions EURO/D1, EURO/D2, UR/D1, UR/D2. Overall, the UR/D1 and the EURO/D2 assumptions match the observed profile rather well, whereas EURO/D1 and UR/D2 seem to fulfil well their expected roles of upper and lower bounds. Thus, if one disregards younger ages, where the proportion of children from 2 to 5 year of age immune to measles in 1996–97 is higher than predicted by the model (perhaps a result of increased routine coverage observed in several Italian areas since mid-1990's), the EURO FOI, as embedded in assumption EURO/D1, does not seem to perform well in the reproducing the seroprevalence profile, in contrast with UR/D1 (e.g. under EURO/D1 the same level of immunity is reached by ages 9–10 years as is reached by the data and UR/D1 by ages 17–19 years).

## Discussion

An attempt has been made to characterise the measles pre-vaccination epidemiological "landscape" in Italy, and also with reference to what we have referred to as the "EURO conjecture" (i.e. that age-selective under-reporting disguises underlying homology with FOIs elsewhere in Europe). There are points open to criticism in this analysis: first, the fact that by looking at "local" forces of infection, e.g. treating spatial sub-areas as autonomous epidemiological units, we disregard spatial correlations between the various local dynamics; this makes the present analysis, at best, a first step. Second, the present work relies only on case reports, the only available pre-vaccination data. Third, the increasing temporal distance between the present time and the pre-vaccination era (ending in 1976) seems to make more and more heroic the assumption that mixing patterns remain unchanged since then. Nevertheless several interesting facts emerge.

### Degree of effort required for eradication at the regional level

Clearly, good data on immunisation coverage and reports of outbreaks of infection would provide some broad picture of patterns of susceptibility. However, as the level of vaccine-based immunity increases, the mean interval between outbreaks also increases so that case reports, even if unbiased, become much less useful as a source of timely information on patterns of susceptiblity. Also in Italy, historically, data on immunisation coverage has been very poor or in many instances completely absent; in many regions case reporting has also been very poor, forming an insensitive tool for detecting small pockets of infection, and large outbreaks, almost by definition, cannot be timely indicators of the presence of susceptibles. Hence the first motivation for the paper was to construct a map of the efforts needed for elimination of measles in Italy, which could then constitute a useful tool for prioritising regional intervention priorities in the context of the agreed WHO target for measles elimination and with the aim of avoiding such outbreaks. During the second half of the 1990's several attempts have been made in Italy to increase routine coverages, which the available data suggested had remained until then disappointingly low (in some regions there have also been campaigns targeted at older age groups, and here it should perhaps be noted that the rationale for such campaigns is re-inforced by the tendency for a lower FOI to increase the accumulation of susceptibles in older age groups which results from sub-optimal vaccination coverage). Even though surveillance data suggests incidence has reached a historic minimum during 1999–2001, national coverage was still well below 70% in 2000, with much regional heterogeneity. It is unsurprising therefore that there have been substantial epidemics causing serious concern, especially in Campania (the region with the lowest coverages) during the spring of 2002, with an estimated 20,000 cases between January and May 2002 [4].

From this standpoint the results here (i.e. basic reproductive ratios etc) suggest that the regions which seem to be the most demanding in terms of effort needed to eradicate the infection are systematically the Southern ones. This is rather problematic, as the Southern regions are those presently characterised by the lowest vaccination coverages [2,3]. The absolute size of the required eradication effort, as summarised by the critical coverages for routine birth vaccination, is somewhat variable and depends on the chosen mixing pattern. For Southern regions critical coverages range from 82%, under "minimally plausible" preferred mixing, to values around 95–97% under realistic assortative mixing, which represent lower and upper bounds for realistic contact patterns. Intermediate assumptions lead to intermediate figures: under "Default" mixing critical coverages for the major Southern Regions (Campania, Puglia, Sicilia) are in the range 86–88%, only two points less than the "EURO" value of 90% [8]. Keeping in mind a) that vaccination at birth is simply a "theoretical standard" (indeed critical coverages quickly increase as the age at vaccination is delayed), b) that we are assuming a 100% effective vaccine, and that, last but not least, c) the strong and persistent presence of anti-vaccination pressure groups in Italy, all these facts indicate that a substantial effort is still needed, especially in the Southern part of the country in order to approach, even in a minimal fashion, the WHO target. A major target of the Italian Measles Elimination Plan currently being initiated will be to strongly reduce regional heterogeneity in vaccine compliance.

### The EURO conjecture

The "EURO conjecture" suggests that the measles FOI in Italy is, in fact, homologous to that observed in other European countries but that, in the case notifications data, this fact is camouflaged by a strong under reporting bias with age.

Under-reporting of childhood diseases has been a major problem in Italy. It is known [7,12] that in the pre-vaccination era the overall reported measles incidence was an order of magnitude less than true incidence, and that the degree of under-reporting varied widely between regions. The existence of large and heterogeneous overall rates of under-reporting at the regional level does not necessarily imply, however, a bias in the (national) estimate of the force of infection: this additionally requires that i) reporting rates exhibit a significant variation with age, or that ii) spatial heterogeneity in overall reporting rates coexist with a marked spatial heterogeneity in infection patterns by age.

**Figure 6:**
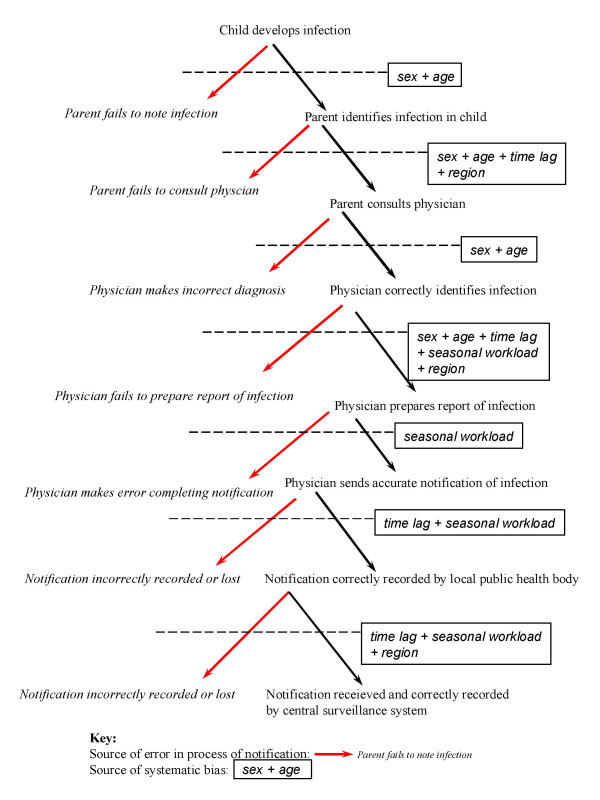
**Potential sources of error in case reporting.** An illustration of potential sources of failure or systematic bias in processes of case notification.

We therefore made an extensive investigation of forces of infection at the spatial level (Regions, but also large conurbations and cities). Our feeling was that by going more deeply spatially it should have been possible to find some "footprint" of the presence of the presumed underlying EURO pattern (e.g. areas with a "high" FOI, or with significant heterogeneity in FOIs). As documented in the Results, we have not been successful in detecting such a "footprint". All "local" forces of infection are systematically substantially smaller compared to the EURO FOI, and surprisingly similar to each other in levels and shape: significant heterogeneity exists only in the youngest age groups. This raises the point, if the EURO conjecture were to be true, of what might be the social processes, if any, underlying the poor age reporting of measles cases, which are capable of so effectively camouflaging the true underlying force of infection. Indeed, in order to appear so stable over space and time, such processes should be profoundly rooted in social responses of families toward diseases of their children and/or medical practitioners' behaviour. In the absence of the relevant data any discussion of the nature of such processes must remain in the realm of speculation, but clearly there is room for bias at many points in the chain leading to reporting of a case [20-22](Fig. 6): perhaps in the historically larger families of Italy because of their size there was more direct family experience of measles and less inclination to seek medical attention when there was an uncomplicated case in the family; perhaps, with measles in young children being a normal stage of life, medical practitioners could have been less inclined to conform to the "bureaucratic" requirements of case reporting; or perhaps there could have been failures in the bureaucratic process itself. Such speculations rely on caricatures of contrasts between Italy and more northern European countries and, clearly, to provide soundly based insights a detailed study would be necessary. Nevertheless, by use of a sentinel survey selective under-reporting by age has indeed been documented in Italy by Ciofi *et al *[23] in recent years for varicella (still in its pre-vaccination period). However their work shows that reporting rates are somewhat higher at young ages (0–10 yr.) and subsequently tend to decrease. Therefore, assuming that current under reporting rates for varicella are in some way similar to those for measles during the pre-vaccination period, their result would not provide evidence in favour of a bias leading to underestimation of the force of infection.

We then investigated the measles incidence time series, encouraged by the fact that [12] reporting rates of measles at the regional level seemed to have remained fairly constant over time, so that (though unreliable in absolute terms) we can reasonably trust that the available incidence data should be broadly representative of the true qualitative patterns. Even in this case, however, we could not find significant footprints of an underlying "higher" force of infection. Rather the results appear to be more or less consistent with the Italian age patterns observed from case reports. Indeed the observed long-term oscillation of measles is around three years or more for most Italian regions. Such figures, though substantially different from the biennial oscillations observed for England & Wales, are by no means uncommon [24]; additionally, compared to England & Wales, they are consistent with a much higher value of the average age at infection, and more generally, with the force of infection. Though one must be cautious about these facts, it is also clear, however, that we cannot quickly dismiss them as the consequence of the presumed existence of age biases in reporting rates: it would mean that patterns of under-reporting are capable of also hiding true time patterns. Though this latter possibility can not be excluded *a priori *(one possibility could be that there is an association between practitioners' reporting rates and the phase of the epidemic cycle, e.g. an epidemic year *vs *a non-epidemic year), it appears rather unlikely in most circumstances. Indeed, if the "true" percentage age distribution of cases is broadly stationary over time, as we would expect in the pre-vaccination era (a fact that Italian data strongly suggest), then significant (and periodic) time changes in age-specific reporting rates would be needed to effectively mask true incidence patterns.

Finally, our modelling work on measles in Italy, shows that the EURO assumption matches rather poorly the serological profile observed for measles in Italy in 1996/7 in the European Sero-epidemiology Network (ESEN) survey [8,25].

Which then was the true force of infection acting in Italy during the pre-vaccination era: the higher EURO FOI, or the lower FOI emerging from Italian case notifications data, or indeed perhaps something in between? Rather than being essentially homologous, as implicit in the EURO conjecture, the possibility that patterns of infection by measles throughout Europe could be and have been largely different (as by the way suggested for rubella by Edmunds *et al*. [26]) is a stimulating one. Our results provide many indications that infection patterns in Italy could indeed deviate from the "EURO" standard. However the uncertainty still present in many factors suggests that more work is urgently needed in order to better understand measles infection patterns in Italy. Be this as it may, we believe that, from a public health perspective, a quite prudent attitude is necessary: knowing that the eradication coverages computed here could just represent lower bounds of the true values, which could be substantially higher, seems to be a sufficient argument for not deviating from the target coverages suggested by WHO. Indeed, the higher *R*_0 _values estimated for the South suggest that, even with achievement uniformly of WHO targets across the regions, elimination of measles will take longer than in the North.

## Conclusions

The results suggest that critical vaccination coverages for elimination are likely to be higher in the south of Italy, precisely where the existing record of coverage is lowest. Substantial efforts are still required if there is to be a realistic hope of achieving WHO targets for measles elimination in Italy, particularly in the south. Notwithstanding this, the evidence does appear to suggest that the force of infection for measles in Italy is indeed somewhat lower than that applicable to other regions participating in the European Sero-Epidemiology Network. More particularly, the evidence suggests that it is probably unlikely that age biases in reporting (suggested elsewhere) could have led to an underestimate of the measles force of infection in Italy.

## Competing interests

The author(s) declare that they have no competing interests.

## Authors' contributions

PM conceived the work, provided the main theoretical analysis and drafted the manuscript, EMC conducted the time series analysis, JRW undertook the modelling work and SS and MCdA contributed epidemiological insight into the Italian public health context of the work. All authors read and approved the final manuscript.
